# Enhancing multi-season wheat yield through plant growth-promoting rhizobacteria using consortium and individual isolate applications

**DOI:** 10.1007/s12223-025-01245-9

**Published:** 2025-02-05

**Authors:** Gerhardus Breedt, Lise Korsten, Jarishma Keriuscia Gokul

**Affiliations:** 1 Limpopo Department of Agriculture and Rural Development, Towoomba ADC, Private Bag X1615, Bela-Bela, 0480 South Africa; 2 Department of Plant and Soil Sciences, University of Pretoria, Private Bag X20, Hatfield, 0028 South Africa; 3 Department of Science and Innovation - National Research Foundation Centre of Excellence in Food Security, Pretoria, Private Bag X20, Hatfield 0028 South Africa; 4 Centre for Microbial Ecology and Genomics, Department of Biochemistry, Genetics and Microbiology, University of Pretoria, Private Bag X20, Hatfield, 0028 South Africa

**Keywords:** Wheat yield enhancement, Reduced fertilizer application, Plant growth-promoting rhizobacteria, Biofertilizers, Whole genome sequencing, Biocontrol

## Abstract

In recent decades, there has been a growing interest in harnessing plant growth-promoting rhizobacteria (PGPR) as a possible mechanism to mitigate the environmental impact of conventional agricultural practices and promote sustainable agricultural production. This study investigated the transferability of promising PGPR research from maize to another *Poaceae* cereal crop, wheat. This multi-seasonal study evaluated the wheat grain yield effect of *Lysinibacillus sphaericus* (T19), *Paenibacillus alvei* (T29) when applied i. individually, ii. as a consortium with *Bacillus safensis* (S7), and iii. at a 75% reduced fertilizer rate. Whole genome sequencing allowed annotation of genes linked to plant growth promotion, providing potential genomic explanations for the observed in-field findings. Application of the consortium compared to a commercial PGPR showed significantly increased wheat yield by 30.71%, and 25.03%, respectively, in season one, and 63.92% and 58.45%, respectively, under reduced fertilizer rates in season two. Individual application of T19 and T29 showed varying results, with T19 increasing wheat yield by 9.33% and 16.22% during seasons three and four but a substantial reduction (33.39%) during season five. T29 exhibited yield increases during season three (9.31%) and five (5.61%) but led to a significant reduction (21.15%) in season four. Genomic analysis unveiled a spectrum of plant growth-promoting genes including those associated with ammonification, phosphate solubilization, ethylene, siderophore, catalase, and superoxide dismutase production. These findings offer valuable insights into the mechanisms behind observed field results, with potential implications for advancing sustainable agriculture and crop productivity in evolving agricultural landscapes.

**Supplementary Information:**

The online version contains supplementary material available at 10.1007/s12223-025-01245-9.

## Introduction

Amidst the challenges posed by a rapidly growing global population and a changing climate, securing sustainable food production has never been more urgent. The Food and Agricultural Organization (Alexandratos and Bruinsma 2012) report “World Agriculture: towards 2015/2030” estimated that 11% of the earth’s surface is presently used for crop production, accounting for approximately 36% of the land being suitable for cultivation, albeit to a limited extent. Conventional agricultural practices, while essential for meeting the world’s dietary demands, often carry a significant environmental toll (Kopittke et al. 2019). Current agricultural strategies are still profoundly reliant on substantial inorganic fertilizer use (Suhag 2016), leading to heavy metal accumulation in the soil, eutrophication, air pollution, soil degradation and increased greenhouse emissions, necessitating the need for conforming to a more sustainable agricultural intensification strategy (Ayala and Rao 2002; Morari et al. 2011; Savci 2012; Smith et al. 2013; Backer et al. 2018; Youssef and Eissa 2014). It is within this crucible of necessity and responsibility that the role of plant growth-promoting rhizobacteria (PGPR) has come to the forefront (Adesemoye and Kloepper 2009; Pareek et al. 2020). This symbiotic solution can not only boost crop yields but also mitigate the ecological consequences of current conventional agricultural practices. This can be achieved by promoting plant health directly, providing nutrients to the plant directly, and/or indirectly, limiting the pathogenic effect on the plant (Backer et al. 2018; Batool and Altaf 2017; Glick 1995; Wu et al. 2009).

The benefits of this technology in agriculture are widely researched but not widely adopted in practical farming operations in South Africa, which can be attributed to inconsistent reproducible results under various “real world” production conditions (Al-Turki et al. 2023). To address the concerns with PGPR inconsistency, Backer et al. (2018) suggested that targeted research be conducted around a singular strain or consortia of limited strains that focus on specific production constraints. In this study, we delve into the promise of PGPR, exploring its potential to revolutionize wheat production and pave the way for a more sustainable agricultural future. Leveraging previous trial results on the application of *Lysinibacillus sphaericus*, *Paenibacillus alvei*, *Bacillus safensis,* *Bacillus pumilus*, and *Brevundimonas vesicularis*, individually and as a consortium on maize (Breedt et al*.* 2017), this novel study aimed to firstly evaluate the plant growth-promoting yield benefits conferred by the previously proven PGPR consortium in another commercially important *Poaceae* grain crop. Secondly, we investigated the efficacy of the PGPR consortium in enhancing wheat yield under conditions of a 75% reduced fertilizer rate, to confirm the fertilizer use efficiency effect previously observed by Batool and Altaf in 2017. Thirdly, we assessed the effect of the most consistent-performing isolates in the Breedt et al. (2017) study individually on wheat over a period of three seasons. Finally, whole genome sequencing was used to identify plant growth-promoting genes of interest in *Lysinibacillus sphaericus* (T19), and *Paenibacillus alvei* (T29) to provide valuable genomic insights into the field research findings.

## Materials and methods

### Maintenance and preparation of PGPR isolates

PGPR cultures for isolates *Lysinibacillus sphaericus* (T19), *Paenibacillus alvei* (T29), and *Bacillus safensis* (S7) were obtained from the University of Pretoria’s PGPR culture collection. These were compared against a commercial PGPR product, Brus® (10^7^ CFU/mL, Stimuplant, Gauteng, South Africa). Isolates were maintained using Microbank™ beads (Pro-Lab Diagnostics, Ontario, Canada), stored at −70°C, and cultured on nutrient agar (Biolab, Wadeville, South Africa) as needed. The isolates were inoculated into sterile nutrient broth (Biolab, Wadeville, South Africa) and incubated for 48 h at 25°C in a shaking incubator. Subsequently, 200 g of sterile Perlite® powder, a granular volcanic rock that acts as an efficient inoculum carrier, was inoculated with 21 mL of 48 h nutrient broth culture (10^6^ CFU/mL) in autoclavable plastic pouches, then incubated for 14 days at ambient temperature to prepare the powder formulation for the respective isolates. For consortium treatment preparation, the individual nutrient broth isolates were added at 1:1 ratios with a total volume of 21 mL per pouch.

### Field trials site description

All field trials were planted at the Towoomba Academic Development Centre (ADC) located on the southern part of the Springbok flats, approximately 4 km southeast of Bela Bela in the Limpopo Province (28°21’E, 24°25’S; 1 184 m above sea level). The trials were planted during autumn (April – May) to ensure the onset of vernalisation during winter (June – August). According to the 50-year average, the long-term daily average minimum and maximum temperatures at Towoomba ADC vary between 3.0 °C and 20.8 °C for July and 29.7 °C and 16.5 °C for December, respectively with an average annual rainfall of 672 mm (Towoomba ADC weather station data). Light frost occurs sporadically during June and July with air temperatures below freezing point for 8 days of the year. The trial areas consisted of a 2 m × 2 m block with a 1.5 m buffer zone around each replicate trial site. Each treatment was replicated three times in a completely randomized design (CRD) and planted by hand in the predominantly Hutton ecotope. To avoid PGPR treatment contamination from the previous season, the trial was moved to untreated soil adjacent to the previous trial plot. Limestone ammonium nitrate (280 g/kg) and superphosphate (10.5%) (Omnia©, Bryanston, South Africa) fertilizer was applied at planting to bring the soil nitrogen and phosphorus level to the required trial design standards of 180 kg/ha nitrogen (N) and 50 kg/ha phosphate (P), respectively, for irrigated wheat (DAFF 2010). Each treatment was prepared by homogenising the Duzi® wheat cultivar seed (Klein Karoo Seed Marketing, Oudtshoorn, South Africa) with 250 g/ha of the inoculated perlite powder prepared as described under “Maintenance and preparation of PGPR isolates”. A consortium of T19, T29, and S7 was applied during the first and second seasons. A separate treatment of the commercial PGPR consortium, Brus®, was also included during the first two seasons for comparative purposes. During the second season of evaluation, N and P fertilizer were reduced to 75% of the recommended fertilizer level. For seasons three, four, and five, isolates T19 and T29 were selected for individual evaluation at standard recommended fertilizer levels as they were the most consistent-performing isolates in Breedt et al. (2017). All seasons included a control treatment where seeds were left untreated but received the same fertilizer rate as the rhizobacterial treatments. Planting density was set at the recommended seeding rate of 120 kg/ha with an inter-row spacing of 15 cm. Trials were irrigated bi-weekly after planting to field capacity until physiological maturity of the wheat crop. Grain yield was collected at a grain content of 12% measured using a moisture analyser (Ohaus MB35, Merck, Darmstadt, Germany) followed by destructive harvesting of the entire 2 × 2 m plot. All trial data was analysed using Proc GLM (general linear model) procedures of SAS 9.4 (Statistical Analysis System, North Carolina, U.S.A) at *P* = 0.05. Means were separated and compared using the Dunnett Least Significant Difference (LSD) test if significant differences were observed.

### Whole genome sequencing, assembly, and annotation

To complement the field trial evaluations and better understand the genetic potential and mechanisms of action of the most promising PGPR isolates, T19 and T29, whole genome sequencing and annotation were conducted to provide deeper insights into their functional genomic capabilities. T19 and T29 were cultured on nutrient agar (Biolab), and the cultures were submitted to the Ion Torrent™ PGM™ Sequencing Facility at the Forestry and Biotechnology Institute (FABI), University of Pretoria, South Africa, for DNA extraction and whole genome sequencing. DNA sequences were generated using the Ion Torrent Personal Genome Machine (PGM) (Life Technologies, Carlsbad, USA) on a 316 chip (400 bp fragment lengths). The sequences were submitted to GenBank under the accession numbers SAMN19982556 and SAMN19982557 for isolate T19 and T29, respectively.

Raw reads were converted to fastq files and assembled de novo into contiguous sequences (contigs) using SeqMan NGen v12 (DNASTAR, Wisconsin, U.S.A) and annotated using the Rapid Annotation Subsystem Technology (RAST) 4.0 server and National Centre for Biotechnology Information (NCBI) prokaryotic genomes annotation pipeline (PGAP). The annotated genomes were viewed in SEED viewer (Overbeek et al. 2014).

## Results

### Impact of PGPR treatment on wheat yield

During the first season, PGPR consortium and Brus® significantly (*P* < 0.001) increased yield (Table 1) by 25.03% and 30.71%, respectively, when compared to the control yield of 5 570 kg/ha. At reduced fertilizer levels in season two, the PGPR consortium treatment and Brus® significantly (*P* = 0.019) increased yield by 63.83% and 58.66%, respectively, when compared to the control yield of 3 290 kg/ha (Online Resource 1).

**Table 1:** The effect of PGPR isolates applied individually and as consortiums on wheat yield over multiple seasons

Yield kg/ha					
**Treatment**	**Season 1**	**Season 2**^**1**^	**Season 3**	**Season 4**	**Season 5**
Control	5 570.72^a^	3 290.00^a^	3 944.20^a^	4 368.52^b^	6 138.89^b^
T19	-	-	4 853.80^b^	4 775.93^b^	4 088.89^a^
T29	-	-	4 311.30^a^	3 444.45^a^	6 483.33^b^
PGPR consortium^2^	6 965.18^b^	5 390.00^b^	-	-	-
Brus® commercial	7 281.23^b^	5 220.00^b^	-	-	-
*P*-value	0.019	< 0.001	0.009	< 0.001	< 0.001
*Cv %*	4.423	2.349	5.411	4.799	7.136
Dunnett test MS value	1 129.7	254.46	552.75	470.75	929.14
^1^ Towoomba trial fertilized at 75% of the recommended 180 kg/ha N and 50 kg/ha P					
^2^ Isolates T19, T29 and S7					
^a,b^ Treatment means within the same column and phosphate level followed by the same letter do not differ significantly, (*P* = 0.05) according to the Dunnetts test					

During the successive seasons, when the isolates were individually applied, strain T19 showed a significant (*P* = 0.009) wheat yield increase of 23.06% during season three, followed by a minor increase of 9.33% in season four, however a significant (*P* < 0.001) yield reduction of 33.39% during season five was noted when compared to the respective control yields. When isolate T29 was considered, wheat yield increased by 9.33% during season three, significantly reduced (*P* < 0.001) by 21.20% during season four, after which it increased by 5.61% in the last season, when compared to respective controls.

### Identification of plant growth-promoting genes in T19 and T29

**Fig. 1:**
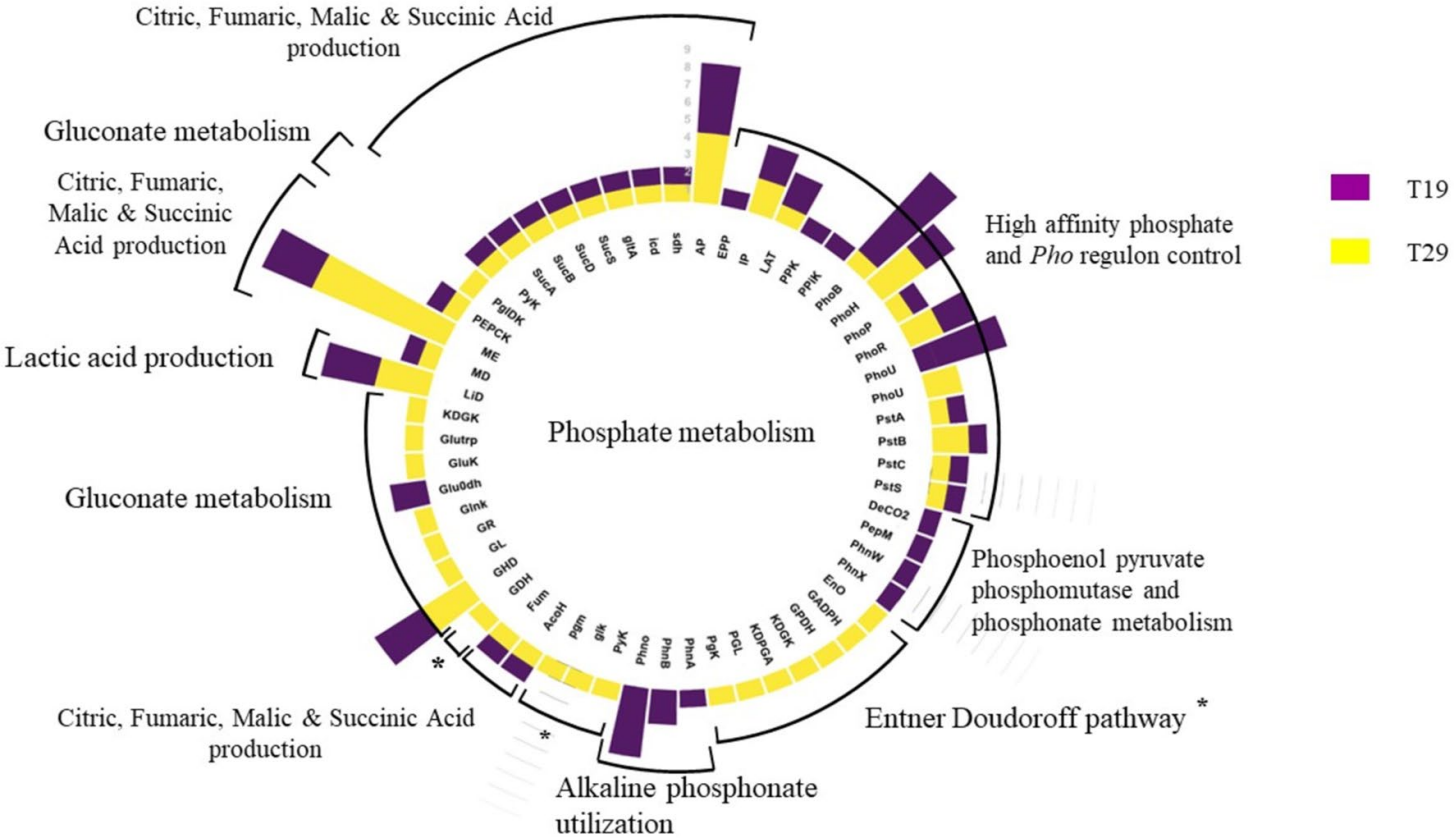
Phosphate metabolism associative genes annotated for T19 and T29

**Fig. 2:**
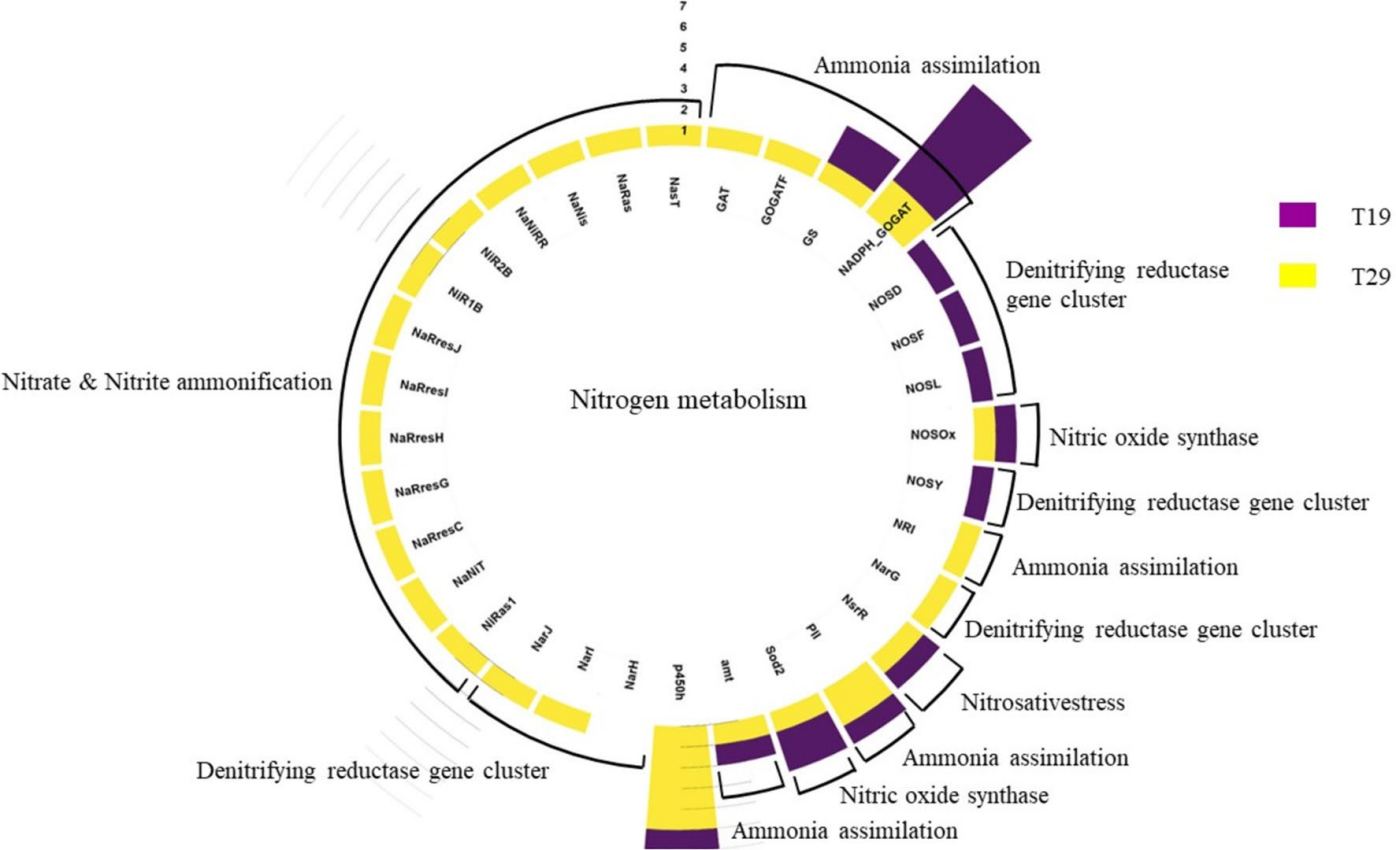
Nitrogen metabolism associative genes identified annotated for isolate T19 and T29

Upon genome sequencing and assembly, the genome size of isolate T19 was determined to be 5 075 018 bp, with a GC content of 36.6%, and yielding 108 contigs. Five thousand and twenty-seven protein-coding sequences (CDS) were assigned, of which 2 045 (41%) were members of the 472 categorized biological subsystems. The PGP genes of interest in the nutrient cycling category were annotated (Online Resource 1), revealing 63 phosphate (Fig. 1) and 19 nitrogen metabolism genes (Fig. 2).

**Fig. 3:**
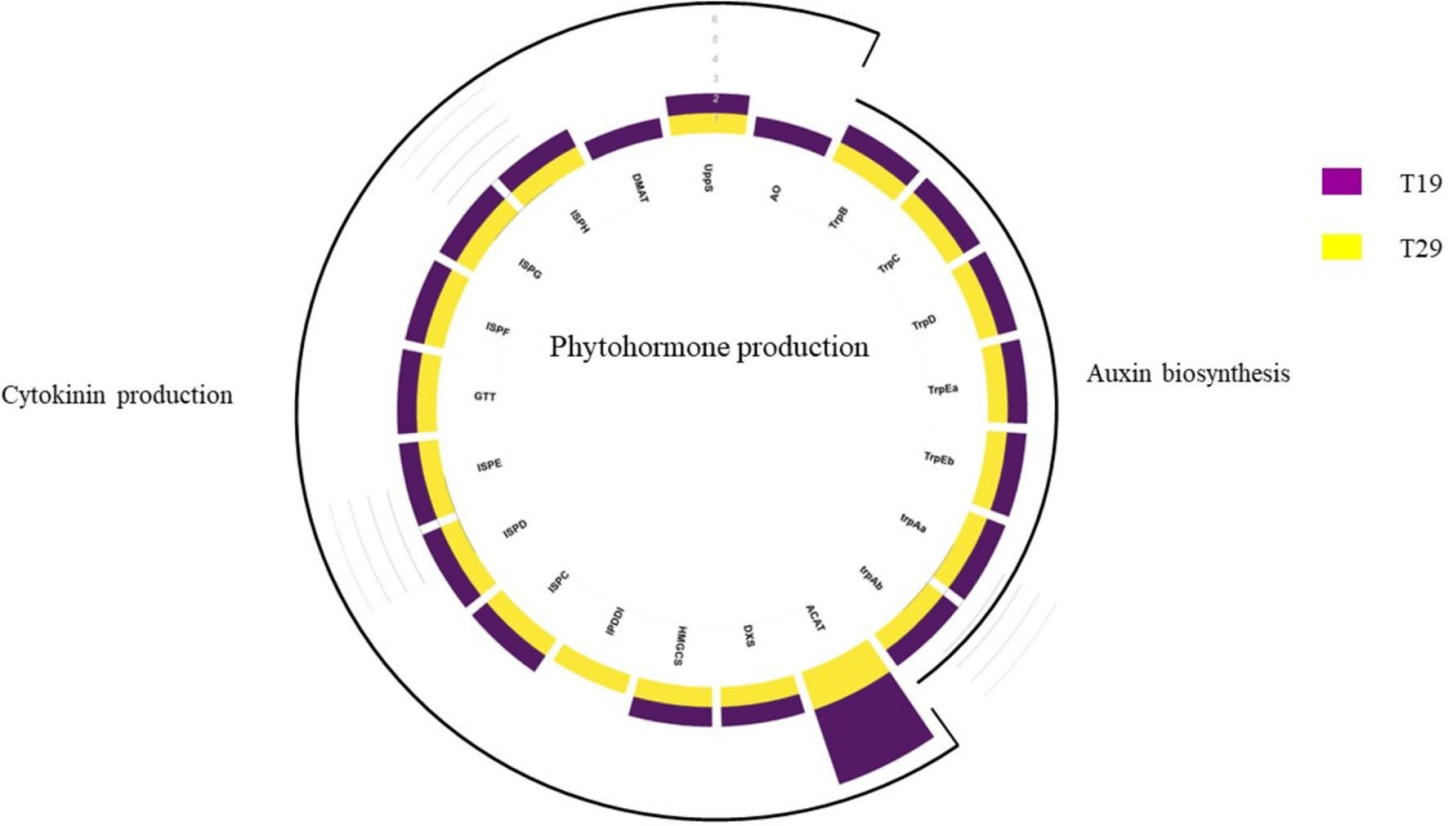
Phytohormone associative genes annotated for T19 and T29

**Fig. 4:**
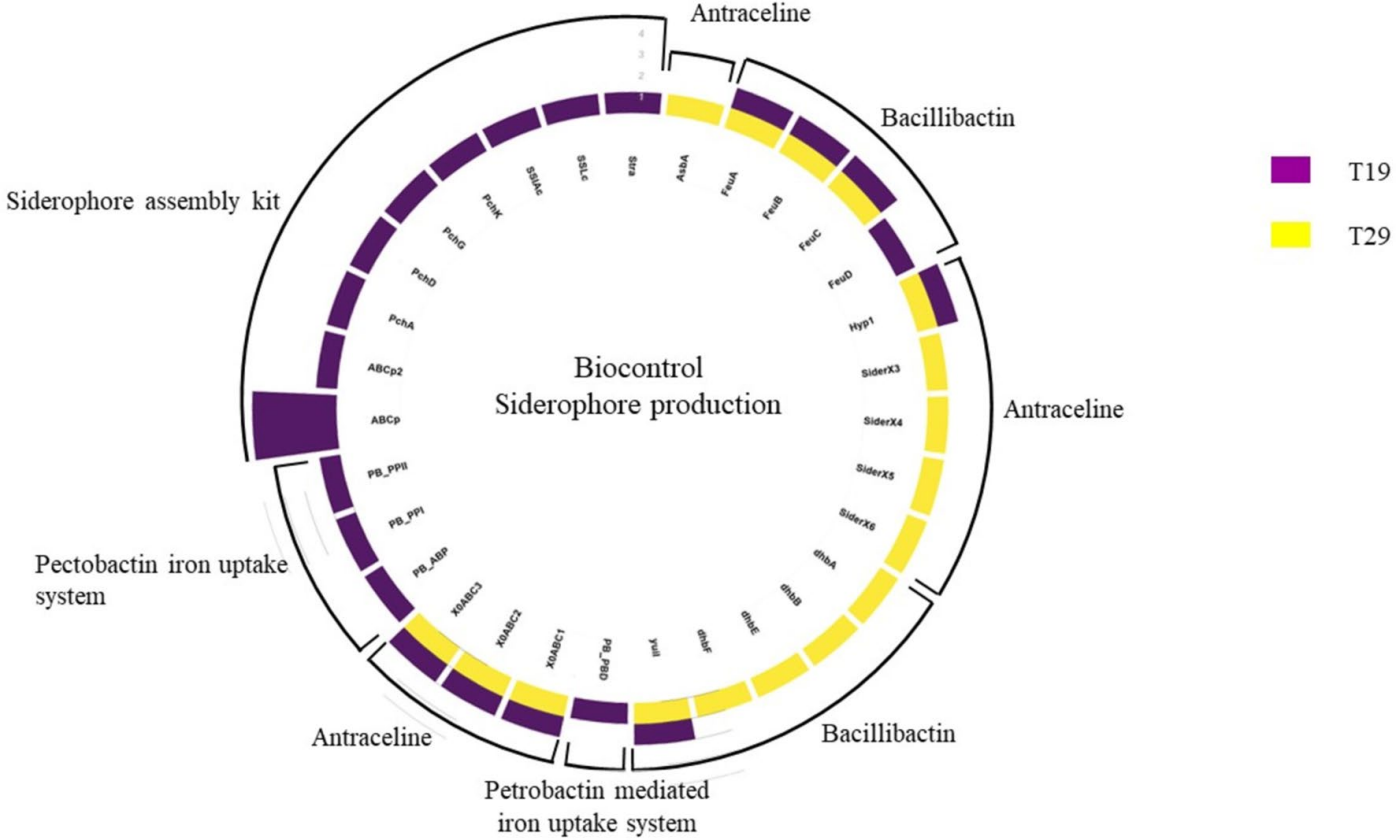
Siderophore associative genes annotated for T19 and T29

In the plant hormone category (Fig. 3), five auxin-producing genes were identified while in the pathogen suppression (Fig. 4) category, 78 genes associated with siderophore production were observed. In the stress regulation category, only eight antioxidant-producing genes were annotated.

Isolate T29 had a genome size of 4 050 096 bp, with a GC content of 43.70%, which yielded 12 contigs. The RAST server assigned 4 205 CDS, of which 2 026 (49%) were members categorized within biological subsystems. In contrast to T19, isolate T29 annotated 30 phosphate, 34 nitrogen metabolism genes, four auxin-producing genes, 13 genes associated with siderophore production, and six antioxidant-producing genes (Online Resource 1).

## Discussion

The positive effects of various PGPR species in the promotion of plant health and improved crop yield under normal production conditions have been well documented in the literature. However, a major challenge associated with PGPR use is the inconsistency in achieving favourable results under field conditions (Gange and Gadhave 2018; Herrmann and Lesueur 2013). This inconsistency can be attributed to variables such as environmental conditions, crop-specific factors, and/or competition with and displacement of the native flora (Martinez-Viveros et al. 2010). Furthermore, not all PGPRs exhibit the same mode of action, making it crucial to consider their suitability for specific production systems and conditions (Choudhary et al. 2011). To address these limitations and enhance the reliability of PGPR applications, current research efforts have shifted towards the use of precise and robust PGPR with a wide spectrum of modes of action (Dessaux et al. 2016).

### PGPR performance in wheat yield field trials

The results from the field trials demonstrate a significant positive impact of PGPR treatment on wheat yield across multiple seasons. During the first season, the consortium of isolates T19, T29, and S7 significantly increased wheat yield at standard recommended fertilizer rates, corroborating earlier findings by Breedt et al. (2017), where these isolates, among others, were evaluated on maize. Likewise, during the second season, when nitrogen and phosphate application rates were reduced, the PGPR consortia continued to show a significant increase in yield aligning with the findings of Batool and Altaf (2017). However, as the study progressed, variations in the performance of individual isolates when applied individually were observed. Isolate T19 significantly increased wheat yield during the third season but showed a decrease in the final season. In contrast, isolate T29 exhibited different dynamics, increasing wheat yield during the third and fifth seasons but reducing wheat yield during the fourth season. A plausible explanation for the season-to-season variations is that the consortia’s presence may lead to niche diversification within the rhizosphere. This diversification enhances microbial diversity, stabilizes the microbiome, and concurrently offers a wide spectrum of PGP mechanisms to support plant growth**.** In its entirety, the results of the current study suggest that the PGPR consortium of T19, T29, and S7 consistently and significantly outperformed individual isolates. This finding aligns with numerous studies including those by Dutta et al. (2018), Liu et al. (2018), Pierson and Weller (1994), Reddy and Saravanan (2013), and Sheirdil et al. (2019), which similarly highlighted the benefits of using PGPR consortia. These studies underscore that a mixture of *Pseudomonas* and *Bacillus* or *Alcaligenes* PGPR isolates tend to enhance crop yield compared to individual isolates. This increase in efficiency is attributed not only to the diverse modes of action conferred by individual isolates but also to the improved survival of applied PGPR within a highly competitive rhizosphere (Dessaux et al. 2016). Furthermore, this study highlights the potential for PGPR application, even under reduced fertilizer application to yield significantly higher crop yields, offering sustainable alternatives for both production and potential ecosystem recovery (Wang et al. 2020). However, it is important to exercise caution, as PGPR applied at fertilizer reductions exceeding 25% of the recommended can result in unstable yields (Batool and Altaf 2017).

### Non-target effects in PGPR application and potential mechanisms of action

Beyond their primary role in improving crop yields, PGPR may have non-target effects. One notable non-target effect of PGPR application is the alteration of the resident microbial communities in the rhizosphere. While the present study primarily focused on the PGPR isolates T19 and T29, their introduction into the rhizosphere may lead to shifts in microbial diversity and composition, which could have far-reaching implications for nutrient cycling, disease suppression, and organic matter decomposition (Moore et al. 2022).

To gain a deeper understanding of the underlying mechanisms of PGPR-mediated wheat yield enhancement, whole genome sequencing and analysis of isolates T19 and T29 (Online Resource 1) were conducted, revealing insights into the potential mechanisms involved. In both isolates, several PGP mechanisms were identified that support the in vitro results. These mechanisms encompass nutrient cycling, phytohormone production, pathogen suppression, and stress regulation.

### Nutrient Cycling Mechanisms

Notably, neither isolate T19 nor T29 was associated with nitrogen fixation (*nif*), indicating a preference for organic nitrogen sources. However, both isolates possessed genes associated with the production of nitric oxide (NO) which can influence plant growth and root development (Agapie et al. 2009; Creus et al. 2005). The additional enzymes, nitrate reductase (*NaR*) and siroheme nitrite reductase (*SiR*) that were annotated for isolate T29 can convert nitrate into plant-absorbable ammonium (Simon 2002).

Another microbial PGP nutrient cycling mechanism is to increase utilizable soil phosphate through mineralization, chelation, or lowering of soil pH (Kalayu 2019). Microbial phosphate solubilization mechanisms are regulated by the two-component phosphonate transport system regulon (*Pho*) which is a global regulatory mechanism that regulates MO phosphate homeostasis (Santos-Beneit 2015). The *Pho* regulon also regulates the translation of extracellular enzymes that can mineralize organic P sources and the associated proteins that store and release these polyphosphate sources. In both isolates, the transporter genes associated with the high-affinity phosphate (*PstS*) and phosphonate transport system (*pho*) were identified that promote the uptake of inorganic P (Pi) in Pi-limited environments by activating phosphate solubilization mechanisms (Brito et al. 2020). The main MO mechanism associated with the solubilization of soil Pi is the secretion of the organic acids e.g. gluconic acid, ∞-keto-gluconic acid, etc. (de Werra et al. 2009). Gluconic acid (GA) can be produced via two pathways; the first is by the enzyme glucose 1-dehydrogenase (*GHD*) which oxidizes glucose with the redox enzyme pyrroloquinoline quinone (PQQ) to form GA. Secondly, GA can be produced when glucose is oxidized to GA and ∞-keto-gluconic acid (KGA) by the enzyme gluconate 2-dehydrogenase to (*Glu-dh*) (Jha et al. 2019). Although the GA-producing enzyme *GHD* was annotated in both isolates, the genes (*pqqABCDEF*) associated with the synthesis of the PQQ-cofactor were not present. Isolate T19 was the only isolate in which the enzyme *Glu-dh* was annotated which according to the findings of Miller et al. (2010) is fundamental in *P. florescens'* ability to solubilize Pi. Although GA production via *Glu-dh* and the PQQ-dependent *GHD* enzymes is well established in literature a study conducted by Leontidou et al. (2020) found that some of their isolates were able to solubilize insoluble Pi on Pikovskaya amended media that lacked *Glu-dh* and the PQQ-dependent *GHD* enzyme with the conclusion that other Pi solubilization mechanisms are available. Vyas and Gulati (2009)stated that organic acids other than the main associated GA and KGA could contribute to Pi solubilization. In both isolates, various organic acid-producing genes other than GA and KGA were annotated, e.g., *LiD*, *PEPCK*, *ME, gltA, AconHn, SucAB, sdh, fum, MD, and gltA* that are associated with lactic acid production, the TCA and reductive TCA cycle (Lui et al. 2022; Wang et al. 2021; Xu et al. 2012). The myriad of organic acid-producing genes annotated could explain the isolate T29's ability to solubilize Pi in the previous study and also support the conclusion made by Leontidou et al. (2020).

Moreover, the presence of genes involved in the breakdown of organic phosphate sources, such as phosphoenolpyruvate phosphomutase (PepM), carboxylase (DeCO2), and 2-aminoethyl phosphonate pyruvate aminotransferase (PhnW), indicates the ability to mineralize organic phosphates, as observed in T19 further contributing to nutrient cycling (Kulakova et al. 2001; Villarreal-Chiu et al. 2012). These mechanisms collectively enhance the availability of essential nutrients to the plant root zone, positively influencing plant growth and yield. Additionally, organic acid production, such as gluconic acid, was noted, which is a well-known mechanism for phosphate solubilization (de Werra et al. 2009).

The capacity of T19 and T29 to enhance soil phosphate solubilization was evident through the occurrence of several genes and various mechanisms, including the high-affinity phosphate (*PstS*) and phosphonate transport system (*pho*), suggesting the potential to enhance phosphate availability in the soil. This capacity to solubilize phosphate compounds is vital for improving nutrient uptake by plants and promoting overall plant growth (Kalayu 2019).

### Phytohormone Regulation

PGPR-mediated phytohormone regulation is another non-target effect that can significantly impact plant growth. Cytokinin production via the non-mevalonate pathway was identified in both isolates, while gibberellin production was absent. Moreover, neither isolate possessed 1-aminocyclopropane-1-carboxylic acid (*ACC*) deaminase, the enzyme responsible for ethylene precursor breakdown. Both isolates exhibited the potential to produce auxins, including the associated genes anthranilate phosphoribosyltransferase (*TrpB*), phosphoribosyl anthranilate isomerase (TrpC), and indole-3-glycerol phosphate synthase (*TrpD*). Auxins play a crucial role in various plant physiological processes, including tropistic responses, root and shoot development, and embryogenesis (Teale et al. 2006). It is essential to maintain optimal hormone levels, as excessive auxin production, e.g., indole 3-acetic acid could have inhibitory effects (Duca et al. 2014). Yet, studies have shown that elevated levels of auxins, may not necessarily adversely affect plant growth (Lobo et al. 2022). Thus, the impact of auxin production by PGPR on plant growth is complex and may depend on various factors, including plant species and environmental conditions.

### Biocontrol and Disease Suppression

Genes associated with siderophore production were observed in isolate T29, including bacillibactin and antrachelin. Siderophores play a crucial role in reducing the bioavailability of iron in the soil, limiting its access to pathogenic microorganisms (Chaiharn et al. 2009; Crowley 2006; Hotta et al. 2010; May et al. 2001; Vargas-Straube et al. 2016). While these siderophores were produced via the non-ribosomal peptide synthetase pathway (NRPS) (Carroll and Moore 2018), the genes for NRPS-independent siderophore (NIS) synthesis were not annotated for either isolate. Furthermore, the transport systems for siderophores, such as the *FeuABC* transporter, were identified in both isolates. The presence of these transport systems suggests a potential strategy for T19 to scavenge iron from siderophore-producing microorganisms providing a competitive advantage within the rhizospere (Gaballa and Helmann 2007).

### Stress Tolerance Mechanisms

The antioxidant-producing enzymes catalase (CAT) and superoxide dismutase (SOD) were also identified in both isolates. These enzymes play a crucial role in mitigating oxidative stress in plants, protecting them from damage caused by reactive oxygen species (ROS) under abiotic stress conditions (Batool et al. 2020). By reducing oxidative stress, PGPR can enhance plant stress tolerance and overall plant health.

The multifaceted mechanisms identified in this study contribute to the observed fluctuations in yield outcomes. Understanding these non-target effects is crucial for harnessing the full potential of PGPR in sustainable agriculture and ecosystem management. These mechanisms should be viewed as interconnected and complementary, but not in isolation, as they often interact and complement each other, emphasizing the complexity of PGPR-mediated plant growth promotion (Kloepper 1993).

## Conclusion

The findings of this study shed light on the impact of PGPR in wheat field trials, with a focus on both consortia and individual isolate applications. The field trial results, demonstrated remarkable increases in yield for the PGPR consortium comprising isolates T19, T29, and S7, corroborating not only the yield increase results of the previous Breedt et al. (2017) study but also validating the transferability of PGPR yield benefits to another *Poaceae* family. Resilience in wheat yield obtained under reduced fertilizer conditions aligns with the broader goal of enhancing fertilizer use efficiency and minimizing environmental impacts. However, the application of individual isolates introduced complexity to our findings, emphasizing the importance of selecting the right combination of PGPR isolates for consortium use to ensure consistent and sustainable crop yield improvement. The various overlapping and synergistic PGP genes identified in both isolates T19 and T29 further support their plant growth-promoting capabilities. These mechanisms (nutrient cycling, phytohormone regulation, biocontrol, and stress tolerance) often work in tandem, creating a dynamic and interconnected network of effects within the rhizosphere. For this technology to be widely adopted in agriculture, field results should be more consistent. To harness the full potential of PGPR, it is imperative to consider their multifaceted effects on nutrient cycling, phytohormone regulation, biocontrol, and stress tolerance, as well as their influence on resident microbiomes. The use of next-generation sequencing technology can provide the platform to effectively identify ‘silent’ PGP mechanisms and tailor PGPR formulations to specific production constraints instead of the older in vitro selection methods. Future research should continue to explore the intricate interplay of these mechanisms, ultimately advancing our understanding of PGPR-mediated plant growth promotion and its role in achieving food security and sustainable agriculture.

## Supplementary Information

Below is the link to the electronic supplementary material.

Supplementary file1 (DOCX 39 KB)

## Data Availability

The whole genome sequence data generated in this study are available in the NCBI repository under accession numbers SAMN 19982556 for T19 and SAMN19982557 for T29. The datasets generated and/or analysed can be made available from the corresponding author upon reasonable request.
