# Supplementary material for: Enhancing multi-season wheat yield through plant growth-promoting rhizobacteria using consortium and individual isolate applications
Source: Folia Microbiol (Praha). 2025 Feb 5;70(6):1295–304. doi: 10.1007/s12223-025-01245-9 (PMC12769497; doi:10.1007/s12223-025-01245-9)
Supplement: Supplementary file 1 — Supplementary file1 (DOCX 39 KB) [file 12223_2025_1245_MOESM1_ESM.docx]

**Enhancing multi-season wheat yield through plant growth-promoting rhizobacteria using consortium and individual isolate applications**

**Supplementary Information**

Gerhardus Breedt^1,2^, Lise Korsten^2,3^ and Jarishma Keriuscia Gokul^2,4^

^1^Towoomba ADC, Limpopo Department of Agriculture and Rural Development, Private Bag X1615, Bela-Bela, 0480, South Africa

^2^Department of Plant and Soil Sciences, University of Pretoria, Private Bag X20, Hatfield, 0028, South Africa

^3^Department of Science and Innovation-National Research Foundation Centre of Excellence in Food Security, South Africa

^4^Centre for Microbial Ecology and Genomics, Department of Biochemistry, Genetics and Microbiology, University of Pretoria, Private Bag X20, Hatfield, South Africa

**Correspondence**: Jarishma Gokul

Tel: +27 12 420 4913; Email: [jarishma.gokul@up.ac.za](mailto:jarishma.gokul@up.ac.za)

Submission to Folia Microbiologica, Springer

Table S1: Gene annotation and genes relating to nutrient cycling, phytohormone production, pathogen suppression, and stress regulation for isolates T19 and T29.

| **PGP mechanism** | **Subsystem feature** | **Trait** | **Annotated gene** | **Gene ID** | **Number of genes** | |
| --- | --- | --- | --- | --- | --- | --- |
|  |  |  |  |  | **T19** | **T29** |
| Nutrient cycling | Phosphate metabolism | High affinity phosphate transporter and control of PHO regulon | Phosphate transport system regulatory protein | *PhoU* | 1 |  |
|  |  |  | Phosphate ABC transporter, periplasmic phosphate binding protein | *PstS* | 1 | 1 |
|  |  |  | Phosphate transport system permease protein | *PstA* | 1 | 1 |
|  |  |  | Phosphate transport system ATP binding protein | *PstB* | 1 | 2 |
|  |  |  | Phosphate transport system permease protein | *PstC* | 1 | 1 |
|  |  |  | Alkaline phosphatase synthesis transcriptional regulatory protein | *PhoP* | 1 | 1 |
|  |  |  | Phosphate regulon sensor protein | *PhoR* | 2 | 2 |
|  |  |  | Phosphate regulon transcriptional regulatory protein | *PhoB* | 6 | 1 |
|  |  |  | Polyphosphate kinase | *PPiK* | 1 | - |
|  |  |  | Inorganic pyrophosphatase | *IP* | 2 | 2 |
|  |  |  | Phosphate starvation inducible protein | *PhoH* | 2 | 3 |
|  |  |  | Alkaline phosphatase | *AP* | 4 | 4 |
|  |  |  | Low affinity inorganic phosphate transporter | *LAT* | 2 | 1 |
|  |  |  | Phosphate regulon sensor protein | *PhoU* | 4 | 2 |
|  |  |  | Polyphosphate kinase | *PPK* | 1 | - |
|  |  |  | Exopolyphosphatase | *EPP* | 1 | - |
|  |  | Phosphoenolpyruvate phosphomutase and phosphonate metabolism | phosphoenolpyruvate phosphomutase | *PepM* | 1 | - |
|  |  |  | Phosphoenolpyruvate decarboxylase | *DeCO2* | 1 | - |
|  |  |  | 2-aminoethylphosphonate pyruvate aminotransferase | *PhnW* | 1 | - |
|  |  |  | Phosphonoacetaldehyde hydrolase | *PhnX* | 1 | - |
|  |  | Alkaline phosphonate utilization | PhnO protein | *Phno* | 4 | - |
|  |  |  | Alkalinephosphonate utilization operon protein | *PhnA* | 1 | - |
|  |  |  | PhnB protein | *PhnB* | 2 | - |
|  |  | Entner doudoroff pathway | Glucokinase | *glk* | - | 1 |
|  |  |  | Glucose-6-phosphate dehydrogenase | *GPDH* | - | 1 |
|  |  |  | 6-phosphogluconolactonase | *PGL* | - | 1 |
|  |  |  | 2-dehydro-3-deoxyphosphogluconate aldolase | *KDPGA* | - | 1 |
|  |  |  | 2-dehydro-3-deoxyphosphogluconate kinase | *KDGK* | - | 1 |
|  |  |  | NAD-dependant glyceraldehyde-3-phosphate dehydrogenase | *GADPH* | - | 1 |
|  |  |  | Phosphoglycerate kinase | *PgK* | - | 1 |
|  |  |  | Phosphoglycerate mutase | *pgm* | - | 1 |
|  |  |  | Enolase | *EnO* | - | 1 |
|  |  |  | Pyruvate kinase | *PyK* | - | 1 |
|  |  |  | Glucose-1-dehydrogenase | *GDH* | - | 1 |
|  |  |  | Gluconolactonase | *GL* | - | 1 |
|  |  |  | Glucokinase | *GluK* | - | 1 |
|  |  | Gluconate metabolism | Glucose-1-dehydrogenase | *GHD* | 3 | 3 |
|  |  |  | Gluconate-2-dehydrogenase | *Glu-dh* | 2 | - |
|  |  |  | Gluconate transcriptional operon suppressor | *GR* | - | 1 |
|  |  |  | Gluconate transporter | *Glc-transporter* | - | 1 |
|  |  |  | Glucokinase | *Glnk* | - | 1 |
|  |  |  | 6-phosphogluconate dehydrogenase decarboxylation | *PgIDK* | - | 1 |
|  |  |  | 2-dehydro-3-deoxygluconate kinase | *KDGK* | - | 1 |
|  |  | Lactic acid | lactate dehydrogenase | *LiD* | 3 | 3 |
|  |  | TCA oxidative and reductive cycle | Pyruvate kinase | *PyK* | 1 | 1 |
|  |  |  | Phosphoenolpyruvate carboxykinase | *PEPCK* | 1 | 1 |
|  |  |  | Malic enzyme | *ME* | 3 | 8 |
|  |  |  | Isocitrate dehydrogenase | *icd* | 1 | 1 |
|  |  |  | Citrate synthase | *gltA* | 1 | 1 |
|  |  |  | Aconitate hydratase | *AcoH* | 1 | 1 |
|  |  |  | 2-oxo-glutarate dehydrogenase E1 component | *SucA* | 1 | 1 |
|  |  |  | 2-oxo-glutarate dehydrogenase E2 component | *SucB* | 1 | 1 |
|  |  |  | Succinyl-CoA ligase alpha chain | *SucD* | 1 | 1 |
|  |  |  | Succinyl-CoA ligase beta chain | *SucS* | 1 | 1 |
|  |  |  | Succinate dehydrogenase | *sdh* | 1 | 1 |
|  |  |  | Fumarate hydratase | *Fum* | 1 | 1 |
|  |  |  | Malate dehydrogenase | *MD* | 1 | 1 |
|  | Nitrogen metabolism | Nitric oxide synthase | Manganese superoxide dismutase | *Sod2* | 2 | 1 |
|  |  |  | Putative cytochrome P450 hydroxylase | *p450h* | 1 | 5 |
|  |  |  | Nitric oxide synthase oxygenase | *NOSOx* | 1 | 1 |
|  |  | Nitrosative stress | Nitrite sensitive transcriptional suppressor | *NsrR* | 1 | 1 |
|  |  | Ammonia assimilation | Glutamine synthetase | *GS* | 2 | 1 |
|  |  |  | Glutamine synthetase chain (large & small) | *NADPH_GOGAT* | 6 | 2 |
|  |  |  | Ferredoxin-dependant glutamate synthase | *GOGATF* | - | 1 |
|  |  |  | Glutamine amidotransferase | *GAT* | - | 1 |
|  |  |  | Nitrogen regulation protein N1 | *NRI* | - | 1 |
|  |  |  | Nitrogen regulation protein P2 | *PII* | 1 | 2 |
|  |  |  | Ammonium transporter | *amt* | 1 | 1 |
|  |  | Denitrifying reductase gene cluster | Nitrous oxide reductase maturation protein outer membrane lipoprotein | *NOSL* | 1 | - |
|  |  |  | Nitrous oxide reductase maturation transmembrane membrane protein | *NOSY* | 1 | - |
|  |  |  | Nitrous oxide reductase maturation protein (ATPase) | *NOSF* | 1 | - |
|  |  |  | Nitrous oxide reductase maturation protein | *NOSD* | 1 | - |
|  |  |  | Respiratory nitrate reductase alpha chain | *NarG* | - | 1 |
|  |  |  | Respiratory nitrate reductase beta chain | *NarH* |  |  |
|  |  |  | Respiratory nitrate reductase delta chain | *NarJ* | - | 1 |
|  |  |  | Respiratory nitrate reductase gamma chain | *NarI* | - | 1 |
|  |  | Nitrate and nitrite ammonification | Nitrite reductase (NADPH large subunit | *NiRas1* | - | 1 |
|  |  |  | Nitrite reductase (NADPH small subunit) | *NiRas2 (NiR1B, Nir2B)* | - | 1 |
|  |  |  | Nitrate/nitrate transporter | *NaNiT* | - | 1 |
|  |  |  | Respiratory nitrate reductase | *NaRres (G,H,I,J,C)* | - | 1 |
|  |  |  | Nitrate/nitrate regulatory/response protein | *Reg (NaNiRR, NaNis, NasT)* | - | 1 |
|  |  |  | Assimilatory nitrate reductase (Large subunit) | *NaRas* | - | 1 |
| Phytohormone production | Auxin production | Auxin biosynthesis | Anthranilate phosphoribosyltransferase | *TrpB* | 1 | 1 |
|  |  |  | Anthranilate synthase aminase component | *trpAa* | 1 | 1 |
|  |  |  | Anthranilate synthase amidotransferase component | *trpAb* | 1 | 1 |
|  |  |  | indole-3-glycerol phosphate synthase | *TrpD* | 1 | 1 |
|  |  |  | Phosphoribosylanthranilate isomerase | *TrpC* | 1 | 1 |
|  |  |  | Tryptophan synthase alpha chain | *TrpEa* | 1 | 1 |
|  |  |  | Tryptophan synthase beta chain | *TrpEb* | 1 | 1 |
|  |  |  | Monoamine oxidase | *AO* | 1 | - |
|  | Cytokinin production | non-mevalonate pathway | Acetyl-CoA acetyltransferase | *ACAT* | 4 | 2 |
|  |  |  | Hydroxymethylglutaryl-CoA synthase | *HMGCS* | 1 | 1 |
|  |  |  | Isopentenyl-diphosphate-delta isomerase | *IPDDI* | - | 1 |
|  |  |  | 1-deoxy-D-xylulose-5-phosphate synthase | *DXS* | 1 | 1 |
|  |  |  | 1-deoxy-D-xylulose-5-phosphate reductoisomerase | *ISPC* | 1 | 1 |
|  |  |  | 2-C-Methyl-D-erythritol 4-phosphate cytidylyltransferase | *ISPD* | 1 | 1 |
|  |  |  | 4-Diphosphocytidyl-2-C-methyl-D-erythritol kinase | *ISPE* | 1 | 1 |
|  |  |  | 2-C-Methyl-D-erythritol 2,4-cyclodiphosphate synthase | *ISPF* | 1 | 1 |
|  |  |  | 1-hydroxy-2-methyl-2-E-buteryl 4-diphosphate synthase | *ISPG* | 1 | 1 |
|  |  |  | 4-Hydroxy-3-methylbut-2-enyl diphosphate reductase | *ISPH* | 1 | 1 |
|  |  |  | (2E,6E)-farnesyl diphosphate synthase | *GTT* | 1 | 1 |
|  |  |  | Undecaprenyl diphosphate synthase | *UppS* | 1 | 1 |
| Pathogen suppression | siderophore production | Bacillibactin siderophore | Dimethylallyltransferase | *DMAT* | 1 | - |
|  |  |  | 2,3-dihydro-2,3-dihydroxybenzoate dehydrogenase | *dhbA* | - | 1 |
|  |  |  | Isochorismatase | *dhbB* | - | 1 |
|  |  |  | 2,3-dihydroxybenzoate-AMP ligase | *dhbE* | - | 1 |
|  |  |  | Bacillibactin synthetase component F | *dhbF* | - | 1 |
|  |  |  | Triactone hydrolase | *yuiI* | 1 | 1 |
|  |  |  | Fe-bacillibactin uptake system (Fe-bacillibactin binding) | *FeuA* | 1 | 1 |
|  |  |  | Fe-bacillibactin uptake system | *FeuB* | 1 | 1 |
|  |  |  | Fe-bacillibactin uptake system | *FeuC* | 1 | 1 |
|  |  |  | Fe-bacillibactin uptake system | *FeuD* | 1 | - |
|  |  | Antrachelin siderophore | Antibiotics biosynthesis monooxygenase domain-containing protein | *Hyp1* | 1 | 1 |
|  |  |  | Anthracheline biosynthesis protein | *AsbA* | - | 1 |
|  |  |  | Anthracheline biosynthesis protein | *AsbA* | - | 1 |
|  |  |  | Long chain fatty acid - CoA ligase | *SiderX3* | - | 1 |
|  |  |  | Protein GBAA1985 associated with anthracheline biosynthesis | *SiderX4* | - | 1 |
|  |  |  | Protein GBAA1986 associated with anthracheline biosynthesis | *SiderX5* | - | 1 |
|  |  |  | Acyl carrier protein associated with anthracheline biosynthesis | *SiderX6* | - | 1 |
|  |  |  | Substrate binding protein in ABC uptake transporter (uncharacterised) | *X-ABC1* | 1 | 1 |
|  |  |  | Permease protein in ABC uptake transporter (uncharacterised) | *X-ABC2* | 1 | 1 |
|  |  |  | ATP binding protein in ABC uptake transporter (uncharacterised) | *X-ABC3* | 1 | 1 |
|  |  | Petrobactin mediated iron uptake system | Petrobactin ABC transporter- periplasmic binding protein | *PB_PBD* | 1 | - |
|  |  |  | Petrobactin ABC transporter- ATP binding protein | *PB_ABP* | 1 | - |
|  |  |  | Petrobactin ABC transporter- permease protein I | *PB_PPI* | 1 | - |
|  |  |  | Petrobactin ABC transporter- permease protein II | *PB_PPII* | 1 | - |
|  |  | Siderophore assembly kit | Siderophore synthetase large component acetyltransferase | *SSIAc* | 1 | - |
|  |  |  | Siderophore synthetase component ligase | *SSLc* | 1 | - |
|  |  |  | Siderophore transfer protein | *Stra* | 1 | - |
|  |  |  | Isochorismate synthase | *PchA* | 1 | - |
|  |  |  | 2,3-dihydroxybenzoate-AMP ligase | *PchD* | 1 | - |
|  |  |  | Thiazolinylimide reductase in siderophore biosynthesis gene cluster | *PchG* | 1 | - |
|  |  |  | Putative reductoisomerase in siderophore biosynthesis gene cluster | *PchK* | 1 | - |
|  |  |  | ABC-type Fe3+ siderophore transport system permease component | *ABCp* | 4 | - |
|  |  |  | ABC-type Fe3+ siderophore transport system permease II component | *ABCp2* | 1 | - |
| Stress regulation | antioxidant activity | Catalase | Catalase | *Cat* | 3 | 5 |
|  |  | Superoxide dismutase | Superoxide dismutase | *sod* | 3 | 3 |
